# The vertical mobility of the first tarsometatarsal joint during demi-plié with forced turnout in ballet dancers

**DOI:** 10.1038/s41598-024-64304-0

**Published:** 2024-07-03

**Authors:** Honoka Ishihara, Noriaki Maeda, Makoto Komiya, Tsubasa Tashiro, Shogo Tsutsumi, Satoshi Arima, Miki Kawai, Yuki Tamura, Yasunari Ikuta, Yukio Urabe

**Affiliations:** 1 Department of Sports Rehabilitation, Graduate School of Biomedical and Health Sciences, Hiroshima University, 1‑2‑3 Kasumi, Minami‑ku, Hiroshima, 734‑8553 Japan; 2 Institute for Human Movement and Medical Sciences, Niigata University of Health and Welfare, Shimami-cho, 1398, Kita-ku, Niigata City, Niigata 950-3198 Japan; 3 Athlete Support Medical Center, Niigata University of Health and Welfare, Niigata, 950-3198 Japan; 4 Department of Orthopaedic Surgery, Graduate School of Biomedical and Health Sciences, Hiroshima University, 1‑2‑3 Kasumi, Minami‑ku, Hiroshima, 734‑8553 Japan; 5 Sports Medical Center, Hiroshima University Hospital, Hiroshima, 1-2-3 Kasumi, Minami-ku, Hiroshima, 734-8553 Japan

**Keywords:** Ballet dancer, Ultrasound, Tarsometatarsal joint, Forced turnout, Musculoskeletal system, Bone, Ligaments, Muscle, Orthopaedics

## Abstract

The forced turnout has a perceived risk of development of hallux valgus (HV) in ballet dancers. We determined how the forced turnout affects the sagittal mobility of the first tarsometatarsal (TMT) joint, which is one of the pathogenic factors of HV development. Seventeen female ballet dancers (body mass index: 18.2 ± 1.8 kg/m^2^) were included and performed demi-plié in control, functional turnout, and forced turnout conditions. Ultrasound imaging synchronized with a three-dimensional motion analysis system was used for measuring the vertical locations of the first metatarsal and medial cuneiform (MC) to evaluate the first TMT joint mobility. Plantar displacement of MC and the first TMT joint mobility in the forced turnout were the greatest among the 3 conditions. Multiple regression analysis indicated that the greater extent of the forcing angle might increase the displacement of MC and the first TMT joint mobility. Evaluating the sagittal mobility of the first TMT joint in the forced turnout can assist in understanding the association between inappropriate techniques including the forced turnout and HV development in ballet dancers. Since the excessive mobility of the first TMT joint is a factor in HV development, the acquirement of adequate active turnout may have the potential to prevent HV development in ballet dancers.

## Introduction

Hallux valgus (HV) is one of the most common forefoot deformities in which with a medial deviation of the first metatarsal and lateral deviation of the great toe which can progress in the long-term^1–5^. Female sex, age, constrictive shoe wear, genetic factors, and first-ray hypermobility are considered pathogenic factors of HV^6–8^. Particularly for classical ballet dancers who have high demand on their forefoot, HV is a serious medical concern due to the pain and its effect on postural balance^7^. However, no evidence to explain clearly that the progression of HV is induced by the continuation of ballet with standing on the tip of the toes and the duration of wearing pointe shoes^5^. A radiographic study revealed that the HV angle (HVA) in both active professional dancers and retired dancers was similar to that of non-dancers, and continuation of ballet does not develop HV^9^.

These previous studies provide us important information that the experience of ballet may be not in itself bad for HV progression. The differences in the extent of risk and its role in the progression of HV need to be considered. Recent studies have described that inappropriate ballet techniques; the forced turnout may involve the development of HV^5,7,10^. Turnout is the external rotation of the lower extremities and a major component of classical ballet position^11^. In contrast, the forced turnout is compensated by the hyper-pronation of the foot and the friction between the plantar surface of the foot and the floor when dancers have less hip joint external rotation enough to make the ideal turnout^12–15^. The forced turnout progresses the subtalar joint pronation and forefoot abduction, resulting in medial longitudinal arch collapse and greater valgus force on the first ray, then increases the risk of HV^13,16,17^. Previous studies reported the effect of forced turnout on the alignment of lower extremities in static posture and the kinematics during ballet movement^7,15^. The forced turnout has no significant effect on the first metatarsophalangeal joint abduction, and no differences were observed between dancers with and without HV. Ballet dancers with HV might have modified the inappropriate techniques which have been performed for the initial stages of their dance careers^15^. Only measuring HVA may be insufficient to evaluate the effect of forced turnout on the pathogenesis of HV.

The first tarsometatarsal (TMT) joint hypermobility is one of the progressive factors of HV due to the laxity of the Lisfranc joint ligament and medial longitudinal arch collapse^3^. Loading on the forefoot with greater valgus force on the first ray in the forced turnout position may affect the mobility of the first TMT joint. In addition, the repetitive vertical movements of the first TMT joint compound the hypermobility of the joint^18,19^.

Recently, a novel system for quantitative evaluation of the first TMT joint mobility has been developed using ultrasound (US) imaging and 3-dimensional motion analysis (MA)^20^. This study has reported the larger mobility of the first TMT joint during gait in women than in men. Generally, women are at higher risk for HV development compared with men^6^, and the results could be interpreted as the mobility in the first TMT joint during movement possibly relates to the risk for HV development. Applying this system can aid to evaluate the dynamics of the first TMT joint and its association with future risk of HV development in dancers. Detailed evaluation of the first TMT joint mobility depending on the forced turnout may assist to clarify the association between inappropriate technique and the risk for HV in dancers. Therefore, the kinematics of the foot during the forced turnout should be elucidated. However, no study has investigated the first TMT joint mobility in dancers. The main purpose of this study was to examine the mobility of the first TMT joint with the forced turnout in dancers and what factors related to the joint mobility. We hypothesized that the first TMT joint mobility is larger in the forced turnout position than in the functional turnout position, and it is associated with the extent of forced turnout and foot pronation.

## Methods

### Participants

In total, 17 female ballet dancers participated in this study (Table 1). All participants have had at least 7 years of experience in classical ballet and had participated in competitions wearing pointe shoes. Seven of the 16 participants had won prizes in competitions at the national level or higher. The exclusion criteria were: (1) ligament injuries, plantar fasciitis, bursitis, or orthopaedic injuries to the hip, knee, ankle, or foot; (2) pain related to hallux valgus; (3) a history of lower extremity surgery; or (4) a score over 10 (highly pronated foot) or − 5 to − 12 (highly supinated foot) on Foot Posture Index-6^21^. The practice time included weekdays and weekends, and the practice time per week was calculated for normal cases, not specifically before competitions or other performances. HVA was evaluated using photographs of the weight-bearing barefoot while standing in a parallel stance. The images were recorded from the knee level, and HVA between the medial aspect of the hallux and the first metatarsal was measured using these images. This method has been reported to be in agreement with radiographic measurement^22^. There was no participant in the present study who was with HV according to a criterion reported in a previous study (HVA > 20°)^23^.

**Table 1:** General characteristics of participants.

Parameters	Mean ± standard deviation
Age (years)	18.1 ± 3.2
Height (cm)	155.2 ± 6.0
Weight (kg)	44.3 ± 6.6
Body mass index (kg/m^2^)	18.2 ± 1.8
Experience (years)	13.3 ± 3.3
Experience with pointe shoes (years)	6.8 ± 3.6
Practice time (hours/week)	17.4 ± 8.8
Practice time with pointe shoes (hours/week)	10.3 ± 5.7
Hallux valgus angle (°)	14.5 ± 3.4
Foot Posture index	3.9 ± 2.6

The study protocols complied with the principles of the Declaration of Helsinki and were approved by the Ethical Committee for Epidemiology of Hiroshima University (approval number: E2021–2702). Informed consent for their participation was obtained from all minor participants and their parents prior to the experiment.

### Experimental tasks

Participants performed standing and demi-plié tasks in three conditions. Demi-plié is a most fundamental and common movement and it is used in most of the other ballet techniques such as rising up to toefunctip standing, or jumping^24^. The details of the three conditions set for this study are given below.

1. Control: Participants stood in the parallel position. According to a previous study, the participants were instructed to stand with their feet 12–14 cm apart, in accordance with the participant’s preference^24^.
2. Functional turnout: Participants were instructed to stand in the first position with a turnout angle determined for each participant. The turnout angle for the functional turnout position was at which each participant could make a turnout without floor friction. Referring to a previous study, the participants were instructed to create a maximum hip joint external rotation in a supine position with an extended hip and knee joint^25^. The physical therapist as an examiner measured the angle between the second toes on each side with respect to the center of the heels of their feet using a goniometer. The measurements were repeated thrice, and the average value was taken as the turnout angle for the functional turnout position.
3. Forced turnout: Participants stood in the first position with a forced turnout angle. This position was in accordance with the previous study^15^ and closer to the first position that participants take in usual ballet classes. Participants had to maintain balance while holding the forced turnout during the trial. The turnout angle was marked at the second toes and the center of their heels with tape on the floor and measured as the angle for this condition.

Both in the functional turnout position and forced turnout position, the participants aligned their feet such that the center of their heels and second toes were on the tape attached to the floor to maintain the same position in each measurement. Additionally, the participants were instructed to stand in a slightly opened position (no heel contact) to not occlude the heel markers. The participants had to maintain their arms at the first position (also known as *En avant*) in ballet to not affect the motion of the pelvis and lower extremities. In the standing task, the participants were instructed to stand in control, functional turnout position, and forced turnout position, and they had to retain the position for a few seconds. In the demi-plié task, the participants performed demi-plié in three conditions according to a 60 bmp metronome beat. The participants were instructed to take 3 s and complete demi-plié with an equal duration of the downward and upward phases. The error in the movement tempo was considered as a failed trial in the experimental task.

### Assessment of the first TMT joint mobility using the US system

The B-mode US system (Art Us EXT-1H, Telemed, Lithuania) with a probe (5–11 MHz, 60 mm field-of-view; Echoblaster, Telemed, Lithuania) was used to evaluate the gap in the location between the first metatarsal and the medial cuneiform in standing, and the motion of the first TMT joint in the sagittal plane during demi-plié^20,26^. The probe was positioned longitudinally over the first TMT joint. The probe position was adjusted finely for each foot to ensure that the first TMT joint was identifiable. Once the probe was placed, the probe was fixed with an elastic band. A US gel pad (Yasojima Proceed Co., Ltd, Japan) was placed between the foot and the probe to improve the quality of images and avoid compression of the skin surface. In accordance with the standardized synchronization system^20^, the US system that captured B-mode US video at 80 fps and 60-mm depth was synchronized with a three-dimensional motion capture analysis system (VICON MX T20-S; Vicon Motion Systems, UK). The beginning of the MA system triggered the simultaneous capture of a B-mode US video. The US videos were visually checked for each trial to ensure that the first metatarsal and medial cuneiform could be marked. When the video was not clear or the probe was misaligned, the measurement was repeated.

### Data acquisition for demi-plié analysis

Participants stood with their feet on two plates (OR-6, 1000 Hz: AMTI, USA), one for each foot. Demi-plié analysis was conducted with the Vicon motion capture analysis system with 16 infrared cameras (100 Hz)^20,27^. The detection of demi-plié motion was processed using the Plug-in-Gait pipeline for Vicon Nexus ver. 1.8.5 software (Vicon Motion Systems, UK). In total, 16 reflective markers were placed on the right and left side of the anterior superior iliac spine, posterior superior iliac spine, thigh, lateral knee joint, lateral lower leg, lateral malleolus, calcaneal tuberosity, and second metatarsal head by the same examiner. The dorsiflexion and pronation angles of the ankle joint were calculated based on the biomechanical model of the Plug-in Gait model^28^. The ankle dorsiflexion angle was measured between the foot vector which is projected into the foot sagittal plane and the sagittal axis of the shank. The foot pronation was measured for an axis perpendicular to the foot vector and the ankle dorsi/plantar flexion axis. It was the angle between the foot vector and the sagittal axis of the shank, projected into the foot transverse plane. We obtained the maximum ankle dorsiflexion angle and the average foot pronation angle during a demi-plié from the time series data.

### Data analysis

**Figure 1:**
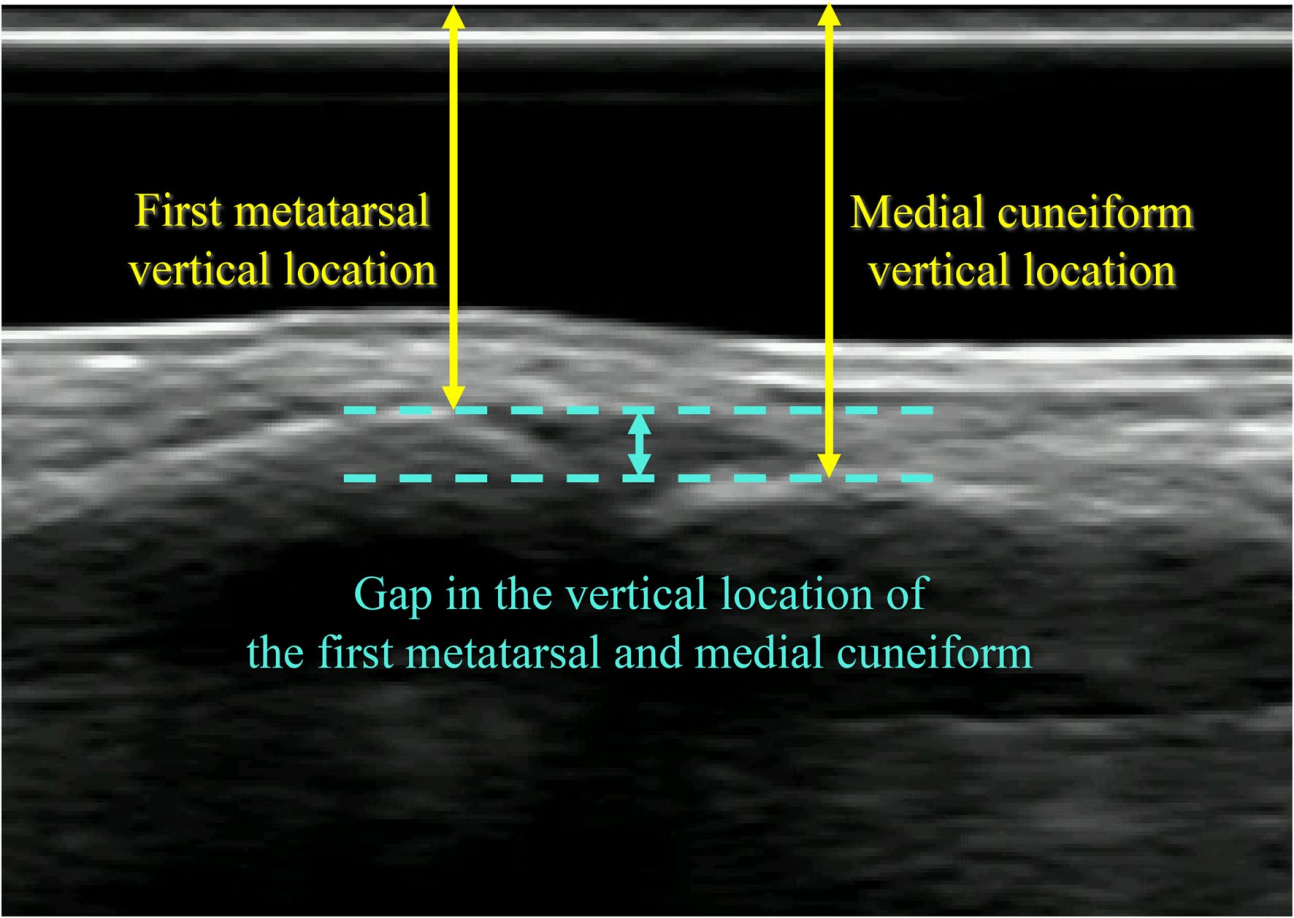
The representative US image. The vertical locations of the first metatarsal and medial cuneiform were determined as the perpendicular distance from the top of the video screen to the dorsal edge of each bone. The gap in the first metatarsal and the medial cuneiform was represented by the vertical distance of the first metatarsal minus that of the medial cuneiform.

Three trials for each condition were captured. The extent of the forcing angle was obtained by subtracting the turnout angle in the functional turnout position from that in the forced turnout position. The phase from the start of knee joint flexion to the end of knee joint extension of the lower extremity attached to the US probe was defined as a demi-plié motion. US video analysis was performed on the detected frames from the beginning to the end of demi-plié. The vertical location and translation of the first metatarsal and medial cuneiform in the US video were calculated with Tracker 5.1.5 software (Open Source Physic, https://www.compadre.org)^29^. As in the previous study, the vertical locations of the first metatarsal and medial cuneiform were determined as the perpendicular distance from the top of the video screen to the dorsal edge of each bone (Fig. 1). The locations of two bones were obtained for all frames corresponding to complete demi-plié. The gap in the first metatarsal and the medial cuneiform was represented by the vertical distance of the first metatarsal minus that of the medial cuneiform. Every shown value of the gap in the first metatarsal and medial cuneiform are represented by the mean value calculated for all participants in each position. The difference in the gap was compared among the three standing tasks. For the demi-plié tasks, the temporal changes in the vertical location of the first TMT joint were analysed by defining the first frame as the neutral level^20^. For the representation of temporal data from three trials in three conditions, one demi-plié analysis duration for each participant was normalized to 100 frames with normalizing software.

### Statistical analysis

Statistical analyses were performed with SPSS Statistics software version 28.0 (IBM Japan Co Ltd, Japan). The normal distribution of all outcomes was confirmed using the Shapiro–Wilk test. Intra-rater reliability for vertical mobilities of the first TMT joint during demi-plié was considered excellent with intraclass correlation coefficients (ICC 1, 3) (Supplementary Information)^30^. Repeated-measures analysis of variance was performed to compare the turnout angle, ankle dorsiflexion, and foot pronation angles during demi-plié, the vertical location of the first metatarsal and medial cuneiform during demi-plié, and the gap in the first metatarsal and medial cuneiform in static stance and during demi-plié. The Bonferroni method was used as a post hoc test among the three conditions. Pearson product-moment correlation test was conducted to detect the correlation coefficients between the US outcomes of demi-plié tasks and other assessed kinematic parameters including the turnout angle, extent of the forcing angle, ankle dorsiflexion, and foot pronation angles. The relationship between the US outcomes and kinematic parameters was examined in a step-wise multiple linear regression. Adjusted R2 was used to estimate the effect size coefficients in the multiple regression analysis. A probability (*p*) value of < 0.05 was considered significant for all statistical tests performed.

## Results

**Figure 2:**
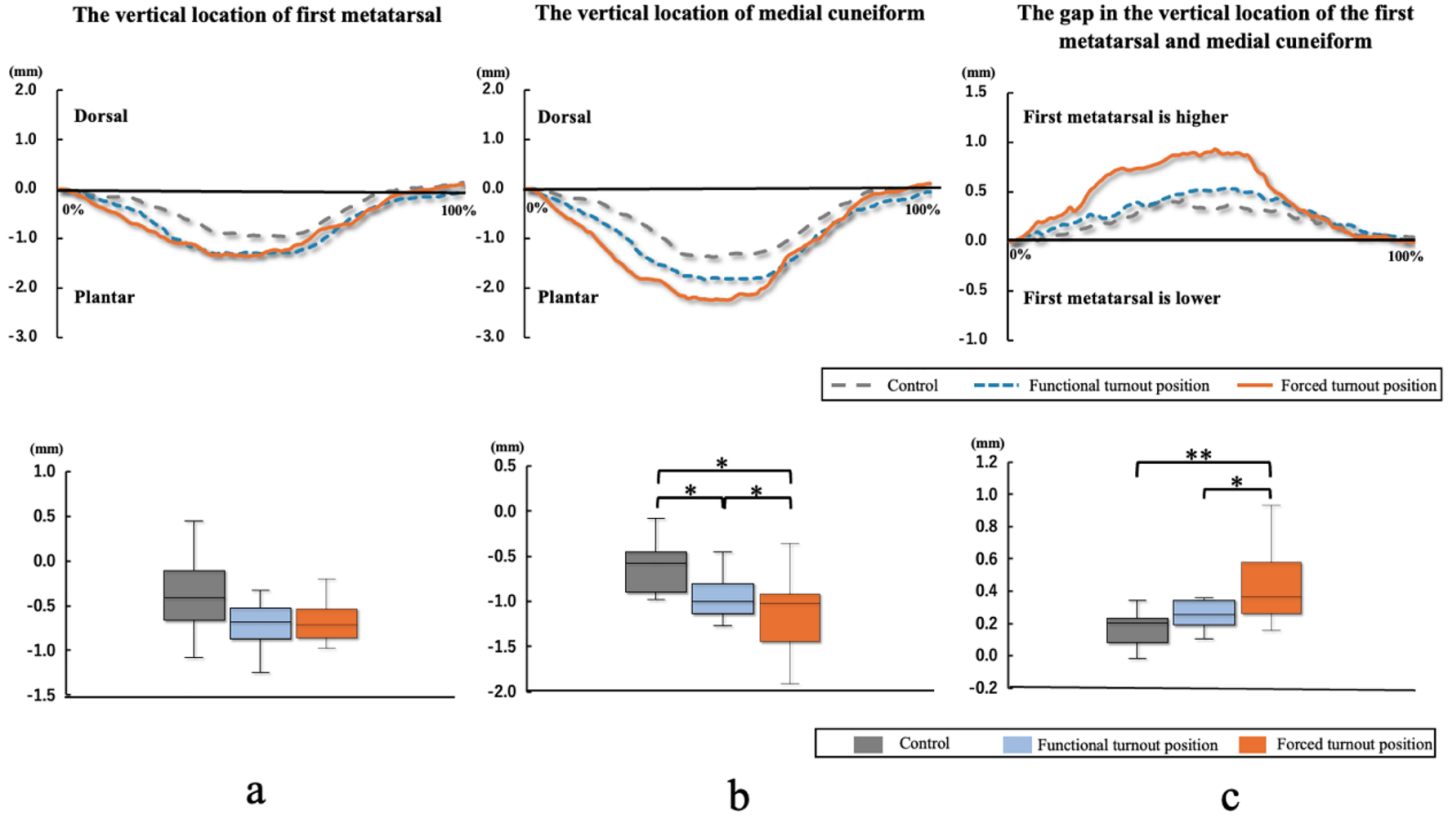
Comparison of the first tarsometatarsal joint movement during demi-plié in three conditions. The graphs on the top row shows data normalized to a demi-plié. Temporal changes in the vertical location of the first metatarsal (**a**) and medial cuneiform (**b**) and the gap in first metatarsal and medial cuneiform (**c**) during demi-plié are shown for the three conditions. *p < 0.05, **p < 0.01.

The comparison of kinematic parameters and US outcomes during static stand and demi-plié in three conditions is shown in Table 2. The turnout angle in the forced turnout position was significantly larger than that in the functional turnout position (*p* < 0.001), and the average extent of the forcing angle was 52.94 ± 13.53°. The maximum ankle dorsiflexion angles during demi-plié in the three conditions were not significantly different. The ankle joint was more pronated during demi-plié in the forced turnout position than that in the control and functional turnout positions (*p* < 0.01). The gap in the first metatarsal and medial cuneiform in the standing task was significantly larger in the forced turnout position than that in the control and functional turnout positions (*p* = 0.048 and *p* = 0.046, respectively). No significant differences were observed in the changes in the vertical location of the first metatarsal during demi-plié among the three conditions (Fig. 2a). The displacement to the plantar direction of medial cuneiform in the forced turnout position was larger than that in the control and functional turnout positions (Fig. 2b; *p* = 0.012 and *p* = 0.046, respectively). The changes in the gap between the first metatarsal and medial cuneiform during demi-plié were significantly larger in the forced turnout position than that in the control and functional turnout positions (Fig. 2c; *p* = 0.009 and *p* = 0.035, respectively).

**Table 2:** Measurement parameters, kinematic data, and US outcomes during statistic stand and demi-plié in three conditions.

Parameters	Control	Functional turnout position	Forced turnout position
Turnout angle (°)	0	112.50 ± 14.89^a^	165.43 ± 4.94^b,c^
Forcing angle (°)	N/A	N/A	52.94 ± 13.53
Maximum ankle dorsiflexion (°)	32.48 ± 4.62	31.10 ± 3.75	28.47 ± 4.83
Average ankle pronation angle (°)	− 1.21 ± 2.83	2.41 ± 2.05^a^	5.02 ± 2.66^b,c^
Gap in first metatarsal and medial cuneiform in standing (mm)	− 0.98 ± 0.53	− 0.99 ± 0.45	− 1.30 ± 0.66^b,c^
Displacement in vertical location of first metatarsal (mm)	− 0.39 ± 0.39	− 0.66 ± 0.32	− 0.68 ± 0.22
Displacement in vertical location of medial cuneiform (mm)	− 0.61 ± 0.59	− 0.95 ± 0.45^a^	− 1.13 ± 0.42^b,c^
Changes of gap in first metatarsal and medial cuneiform (mm)	0.20 ± 0.22	0.28 ± 0.14	0.45 ± 0.25^b,c^

All data were represented with mean and standard deviation.

N/A, not applicable.

^a^*p* < 0.05 between control and reasonable first positions.

^b^*p* < 0.05 between control and forced first positions.

^c^*p* < 0.05 between reasonable and forced first positions. The angle of forced turnout was equal to the maximum active turnout angle and calculated by subtracting the turnout angle in the reasonable first position from that in the forced turnout position.

Table 3 summarizes the correlation for the kinematic parameters of the first TMT joint during demi-plié and measurement parameters. The turnout angle in the functional turnout position, which means the maximum active turnout angle showed a significantly strong correlation with the displacement in the vertical location of the medial cuneiform (r = 0.740, *p* = 0.014). The turnout angle in the functional turnout position also had a significantly strong negative correlation with the changes in the gap in the first metatarsal and medial cuneiform (r = − 0.728, *p* = 0.017). The turnout angle for the functional turnout position, which is suggested the maximum active hip joint external rotation of each participant, showed significant correlations with the changes in the vertical location of the medial cuneiform (r = 0.740, p = 0.014) and in the gap in the first metatarsal and medial cuneiform (r = -0.728, p = 0.017). The extent of the forcing angle showed significant strong correlations with the changes in the vertical location of the medial cuneiform (r = 0.779, *p* = 0.008) and in the gap in the first metatarsal and medial cuneiform (r = 0.775, *p* = 0.008). There were significant moderate correlations between both maximum ankle dorsiflexion and the average foot pronation angle, and the changes in the vertical location of the medial cuneiform (r = 0.658, *p* = 0.009 and r = 0.515, *p* = 0.039, respectively). Table 4 presents the results of multiple regression analysis of the associations of US measurements during demi-plié in the forced turnout position with measurement parameters. Only the extent of the forcing angle is associated with the vertical displacement in the medial cuneiform and the changes in the gap in the first metatarsal and medial cuneiform (β = 0.779, *p* = 0.008, R^2^ = 0.557 and β = 0.775, *p* = 0.008, R^2^ = 0.551, respectively).

**Table 3:** Correlation analysis between differences in the first metatarsal, medial cuneiform, and gap during demi-plié and measurement parameters.

	First metatarsal (mm)						Medial cuneiform (mm)						Gap in first metatarsal and medial cuneiform (mm)					
	Control		Functional turnout position		Forced turnout position		Control		Functional turnout position		Forced turnout position		Control		Functional turnout position		Forced turnout position	
	r	*p*	r	*p*	r	*p*	r	*p*	r	*p*	r	*p*	r	*p*	r	*p*	r	*p*
Turnout angle																		
Functional turnout position	0.394	0.260	0.392	0.263	0.597	0.068	0.351	0.320	0.462	0.179	**0.740**	**0.014**	-0.312	0.381	− 0.571	0.085	− **0.728**	**0.017**
Forced turnout position	0.168	0.642	0.088	0.809	0.375	0.286	0.090	0.804	0.133	0.715	0.396	0.257	0.032	0.930	− 0.220	0.542	-0.341	0.334
The forcing angle	− 0.423	0.223	0.435	0.209	0.619	0.056	0.388	0.268	0.507	0.135	**0.779**	**0.008**	0.365	0.300	0.617	0.057	**0.775**	**0.008**
Ankle angle during *plié*																		
Maximum ankle dorsiflexion angle	− 0.055	0.879	− 0.408	0.242	0.622	0.055	0.000	0.999	-0.294	0.409	**0.658**	**0.009**	− 0.077	0.833	0.004	0.992	− 0.568	0.087
The forcing angle	0.246	0.493	0.224	0.535	0.463	0.178	0.180	0.618	0.222	0.538	**0.515**	**0.039**	0.107	0.769	0.193	0.592	0.466	0.075

Significant correlations are in bold.

**Table 4:** Multiple regression analysis.

Assessed parameter	Medial cuneiform of *plié* in forced first position (mm)					Gap in first metatarsal and medial cuneiform of *plié* in forced turnout position (mm)				
	β	95% CI		*p*	*R*^*2*^	β	95% CI		*p*	*R*^*2*^
		Lower	Upper				Lower	Upper		
Forcing angle	0.779	0.027	0.006	0.008	0.557	0.775	0.003	0.016	0.008	0.551

Explanatory variable was the forcing angle which was calculated by subtracting the turnout angle of the functional turnout position from the turnout angle of the forced turnout position.

*β* standardized partial regression coefficient, *CI* confidence interval.

## Discussion

To the best of our knowledge, this is the first study to clarify the mobility of the first TMT joint in the sagittal plane during demi-plié among different turnout positions in dancers who are specific in ballet using a synchronized US and MA system. The changes of vertical location in the medial cuneiform and the gap in the TMT joint during demi-plié were greater in the forced turnout position than those in the control and functional turnout positions. Further, the extent of the forcing angle was associated with the vertical displacement in the medial cuneiform and the changes in the gap between the first metatarsal and medial cuneiform. Our study captured the differences in the first TMT joint dynamics during demi-plié depending on the first position, suggesting the effect of forced turnout on increasing the hypermobility of the first TMT joint.

Correct turnout is initiated and mainly undertaken by the hip joint^31^. The contribution of each lower-extremity joint to turnout, 50–70% of external rotation comes from the hip joint, and the other 30–50% is covered with knee, ankle, and foot^32,33^. The reduction of hip external rotation in turnout standing results in compensation at the ankle and foot^12–14^. It is important for demi-plié to maintain all parts of the plantar foot on the floor continuously, maintaining the sustained arch, with the toes stretched out and pressing down on the floor^34^. A collapse of the medial longitudinal arch and excessive foot pronation were induced by the foot hyperabduction to maintain standing in the forced turnout position^35^. The rotation force created from the floor, when dancers stand in forced turnout via the friction between the feet and floor, is conceived to make the tibia internally rotated relative to the foot and subtalar joint pronation^13^, resulting in the first TMT joint unlocking^15^. This mechanism allows forefoot abduction^15^, and the medial longitudinal arch collapse^13,16,17^. The collapsing medial longitudinal arch has a relation with the overload of the midfoot and the hypermobility of the first TMT joint^8^. The present study revealed that demi-plié in the forced turnout position, the excessive turnout position over the maximum active turnout angle, leads to the plantar displacement of the medial cuneiform and the larger gap in the first TMT joint. This finding suggests that forced turnout possibly underlies the hypermobility of the first TMT joint and midfoot collapse^8^, and repetitive loading with forced turnout may contribute to the development of the HV deformity in dancers through the hypermobility of the first TMT joint^18,19^.

The turnout angle in the functional turnout position has a negative correlation with the gap between the first metatarsal and medial cuneiform in the forced turnout position. The multiple regression analysis showed the extent of the forcing angle is associated with the changes in the gap in the first TMT joint. The dancers who have a small active turnout angle cannot avoid the extent of the forcing angle to keep the forced turnout position, and the active turnout angle should be improved to prevent compounding hypermobility in the first TMT joint. Demi-plié is one of the most basic skills and is used in majority of the ballet techniques^7^, and our results in demi-plié can be generalized to various techniques in ballet. The foot pronation angle did not show a significant association with the changes in the gap in the first metatarsal and medial cuneiform. This may be attributed to small loads on foot by demi-plié motion. Previous studies described that the medial longitudinal arch was lower in dynamic movements such as *sautés* (a small jump of ballet) than in standing or demi-plié among different turnout positions^15,36^. Therefore, the higher correlation between the mobility of the first TMT joint and measurement parameters including the extent of the forcing angle and foot pronation may be found in more dynamic tasks.

The present study has several limitations. First, the US measurements obtained in this study evaluated only the vertical mobility of the first TMT joint. Although this study focused on the sagittal plane based on the report that the main elements of motion of the first metatarsal are likely to be placed in the sagittal plane^18^. Whether the joint instability in the coronal plane may affect the HV or not has never been reported, the possibility need to be investigate in the future. Further, the possibility that the some variability in observed differences is related to actual changes in relative US probe placement between participants cannot be dismissed. It is necessary to improve the methods for evaluating the detailed movement of the foot and the association between the lower extremities and foot movements and the gap in the first TMT joint. Second, this study was conducted with barefoot. In classical ballet, dancers wear ballet shoes (flat shoes) or pointe shoes. Constricted forefoot or tying pointe shoes’ ribbon around the ankle joint may affect the movements and mobility of each joint. Finally, this is a cross-sectional study, and the relationship between the captured hypermobility in the first TMT joint and HV incidence is unclear. Further longitudinal studies are needed to investigate the association with the TMT joint mobility and the incidence of HV.

In conclusion, the first TMT joint mobility during demi-plié was greater in the forced turnout position than in the functional turnout position, and the extent of the forcing angle was associated with the first TMT joint mobility. Our results suggest that the forced turnout may contribute to hypermobility in the first TMT joint which is one of the risk factors of HV. Therefore, the association between forced turnout and the first TMT joint mobility should be focused on, and increasing the active turnout angle, namely decreasing the gap between active and forced turnout angles, may be important to prevent developing HV in ballet dancers.

### Supplementary Information

Supplementary Information.

## Data Availability

The datasets for the present study are available upon request from the corresponding author.

## References

[1] Coughlin MJ, Jones CP (2007). Hallux valgus: Demographics, etiology, and radiographic assessment. Foot. Ankle. Int..

[2] Dykyj D (1989). Pathologic anatomy of hallux abducto valgus. Clin. Podiatr. Med. Surg..

[3] Glasoe WM, Nuckley DJ, Ludewig PM (2010). Hallux valgus and the first metatarsal arch segment: A theoretical biomechanical perspective. Phys. Ther..

[4] Piqué-Vidal C, Vila J (2009). A geometric analysis of hallux valgus: Correlation with clinical assessment of severity. J. Foot. Ankle. Res..

[5] Davenport KL, Simmel L, Kadel N (2014). Hallux valgus in dancers: A closer look at dance technique and its impact on dancers’ feet. J. Dance. Med. Sci..

[6] Perera AM, Mason L, Stephens MM (2011). The pathogenesis of hallux valgus. J. Bone. Joint. Surg. Am..

[7] Seki H, Miura A, Sato N, Yuda J, Shimauchi T (2020). Correlation between degree of hallux valgus and kinematics in classical ballet: A pilot study. PLoS. One..

[8] Biz C, Favero L, Stecco C, Aldegheri R (2013). Hypermobility of the first ray in ballet dancer. Muscles. Ligaments. Tendons. J..

[9] Einarsdóttir H, Troell S, Wykman A (1995). Hallux valgus in ballet dancers: A myth. Foot. Ankle. Int..

[10] Howse J (1983). Disorders of the great toe in dancers. Clin. Sports. Med..

[11] Quanbeck AE, Russell JA, Handley SC, Quanbeck DS (2017). Kinematic analysis of hip and knee rotation and other contributors to ballet turnout. J. Sports. Sci..

[12] Coplan JA (2002). Ballet dancer’s turnout and its relationship to self-reported injury. J. Orthop. Sports. Phys. Ther..

[13] Nowacki RM, Air ME, Rietveld AB (2012). Hyper-pronation in dancers incidence and relation to calcaneal angle. J. Dance. Med. Sci..

[14] Hendry D, Campbell A, Ng L, Grisbrook TL, Hopper DM (2015). Effect of Mulligan’s and Kinesio knee taping on adolescent ballet dancers knee and hip biomechanics during landing. Scand. J. Med. Sci. Sports..

[15] Carter SL, Bryant AR, Hopper LS (2019). An analysis of the foot in turnout using a dance specific 3D multi-segment foot model. J. Foot. Ankle. Res..

[16] Ahonen J (2008). Biomechanics of the foot in dance: A literature review. J. Dance. Med. Sci..

[17] Conti S, Wong Y (2001). Foot and ankle injuries in the dancer. J. Dance. Med. Sci..

[18] Allen MK (2004). Relationship between static mobility of the first ray and first ray, midfoot, and hindfoot motion during gait. Foot. Ankle. Int..

[19] Glasoe WM, Coughlin MJ, Morton DJ (2006). A critical analysis of Dudley Morton’s concept of disordered foot function. J. Foot. Ankle. Surg..

[20] Maeda N (2022). Quantitative evaluation of the vertical mobility of the first tarsometatarsal joint during stance phase of gait. Sci. Rep..

[21] Redmond AC, Crosbie J, Ouvrier RA (2006). Development and validation of a novel rating system for scoring standing foot posture: The Foot Posture Index. Clin. Biomech. Bristol. Avon..

[22] Janssen DM, Sanders AP, Guldemond NA, Hermus J, Walenkamp GH, van Rhijn LW (2014). A comparison of hallux valgus angles assessed with computerised plantar pressure measurements, clinical examination and radiography in patients with diabetes. J. Foot. Ankle. Res..

[23] Piqué-Vidal C, Solé MT, Antich J (2007). Hallux valgus inheritance: Pedigree research in 350 patients with bunion deformity. J. Foot. Ankle. Surg..

[24] Schrefl A, van de Langenberg R, Schärli A (2021). Kinematic analysis of the coupling between calcaneal eversion and ankle dorsiflexion in a contemporary dancer’s demi-plié. Med. Probl. Perform. Art..

[25] Champion LM, Chatfield SJ (2008). Measurement of turnout in dance research. A critical review. J. Dance. Med. Sci..

[26] Rosa LG, Zia JS, Inan OT, Sawicki GS (2021). Machine learning to extract muscle fascicle length changes from dynamic ultrasound images in real-time. PLoS. One..

[27] Ishii Y (2020). Dynamic ultrasonography of the medial meniscus during walking in knee osteoarthritis. Knee.

[28] Vicon Motion Systems Ltd. Plug-in Gait Reference Guide. Available online: https://docs.vicon.com/display/Nexus25/Plug-in+Gait+kinematic+variables (2023)

[29] Brown D, Cox AJ (2009). Innovative uses of video analysis. Phys. Teach..

[30] Koo TK, Li MY (2016). A guideline of selecting and reporting intraclass correlation coefficients for reliability research. J. Chiropr. Med..

[31] Gilbert CB, Gross MT, Klug KB (1998). Relationship between hip external rotation and turnout angle for the five classical ballet positions. J. Orthop. Sports. Phys. Ther..

[32] Sherman AJ, Mayall E, Tasker SL (2014). Can a prescribed turnout conditioning program reduce the differential between passive and active turnout in pre-professional dancers. J. Dance. Med. Sci..

[33] Gorwa J, Kabaciński J, Murawa M, Fryzowicz A (2020). On the track of the ideal turnout: Electromyographic and kinematic analysis of the five classical ballet positions. PLoS. One..

[34] Gontijo KN, Candotti CT, Feijó GD, Ribeiro LP, Loss JF (2015). Kinematic evaluation of the classical ballet step “plié”. J. Dance Med. Sci..

[35] Ryan AJ, Stephens RE (1987). Dance medicine: a comprehensive guide.

[36] de Mello Viero CC (2017). Height of the medial longitudinal arch during classical ballet steps. J. Dance. Med. Sci..

