# Supplementary material for: The vertical mobility of the first tarsometatarsal joint during demi-plié with forced turnout in ballet dancers
Source: Sci Rep. 2024 Jul 3;14:15321. doi: 10.1038/s41598-024-64304-0 (PMC11222447; doi:10.1038/s41598-024-64304-0)
Supplement: Supplementary file 1 — Supplementary Information. [file 41598_2024_64304_MOESM1_ESM.docx]

The vertical mobility of the first tarsometatarsal joint during demi-plié with forced turnout in ballet dancers

Honoka Ishihara^1^, Noriaki Maeda^1^, Makoto Komiya^2^, Tsubasa Tashiro^1^, Shogo Tsutsumi^1^, Satoshi Arima^1^, Miki Kawai^1^, Yuki Tamura^1^, Yasunari Ikuta^3, 4^,Yukio Urabe^1*^

***Supplementary Information***

- Determining the functional turnout and forced-turnout positions.

  **Figure**. The method of determining functional turnout and forced-turnout positions. Referring to a previous study, the participants were instructed to create the maximum hip-joint external rotation in a supine position with an extended hip and knee joint. The angle between the second toes on each side with respect to the centre of the heels of their feet was measured (i) using a goniometer and was used to determine the angle for the functional turnout position (**a**). The forced-turnout position was closer to the first position that participants take in usual ballet classes (**b**). When participants maintain the forced-turnout angle (ii), using the friction between the floor and plantar area of foot or foot hyper-pronation in this position was allowed.
- The setting of the probe on foot.

**Figure**. The placement of US probe. The probe was positioned longitudinally over the first tarsometatarsal (TMT) joint. A US gel pad was placed between the foot and the probe to improve the quality of images and avoid compression of the skin surface.

- Intra-rater reliability for vertical mobilities of the first tarsometatarsal (TMT) joint during demi-plié was considered excellent in the location of the metatarsals and medial cuneiform, and the gap of the first metatarsal and medial cuneiform. ICC 1,3 was regarded as excellent if more than 0.74, good between 0.60 to 0.74, fair between 0.40 to 0.59, and poor on less than 0.4032) [32].

**Table**. Reproducibility for the US measurements in the vertical mobilities of the first metatarsal joint

|  | **Control** | | |  | **Reasonable first position** | | |  | **Forced first position** | | | |
| --- | --- | --- | --- | --- | --- | --- | --- | --- | --- | --- | --- | --- |
| **Temporal data** | **ICC (1,3)** | **95% CI** | |  | **ICC (1,3)** | **95% CI** | |  | **ICC (1,3)** | **95% CI** | |  |
|  |  | **Lower** | **Upper** |  |  | **Lower** | **Upper** |  |  | **Lower** | **Upper** |  |
| **First metatarsal** | 0.863 | 0.825 | 0.885 |  | 0.852 | 0.809 | 0.917 |  | 0.842 | 0.829 | 0.907 |  |
| **Medial cuneiform** | 0.809 | 0.781 | 0.821 |  | 0.769 | 0.740 | 0.885 |  | 0.769 | 0.754 | 0.858 |  |
| **Gap in first metatarsal and**  **medial cuneiform** | 0.823 | 0.781 | 0.852 |  | 0.771 | 0.749 | 0.840 |  | 0.746 | 0.737 | 0.840 |  |

ICC, intraclass correlation coefficient; CI, confidence interval.
